# Human Metapneumovirus Associated With Acute Hemorrhagic Oedema of Infancy: A Case Report

**DOI:** 10.7759/cureus.73296

**Published:** 2024-11-08

**Authors:** Kuldeep Dhariwal

**Affiliations:** 1 Paediatrics, NMC Specialty Hospital, Dubai, ARE

**Keywords:** acute haemorrhagic oedema of infancy (ahoi), cutaneous leukocytoclastic vasculitis, cytomegalovirus (cmv) infection, human metapneumovirus (hmpv) infection, pediatric infectious diseases

## Abstract

Acute Haemorrhagic Oedema of Infancy (AHOI), also known as Acute Hemorrhagic Edema of Infancy (AHEI), is characterized by purpuric skin lesions, edema, and fever. It is classified as a form of cutaneous leukocytoclastic vasculitis. Clinically, AHOI presents with targetoid, purplish spots on the face and limbs, accompanied by the sudden onset of peripheral edema. AHOI is often associated with infections, including rotavirus, cytomegalovirus (CMV), and pneumococcal bacteremia. In this case, an eight-year-old male infant presented to the emergency room with fever, cough, and a newly developed, non-blanching red macular rash that spread across his face, neck, chest, abdomen, back, and legs, gradually increasing in size over the course of illness. A complete blood count revealed neutrophilia with a normal leukocyte count, and C-reactive protein levels were elevated at 70 mg/L. Human metapneumovirus (hMPV) was detected in a nasopharyngeal aspirate. Abdominal ultrasound showed moderate splenomegaly and enterocolitis. This case highlights a rare association between AHOI and hMPV infection, contributing to the understanding of hMPV as a potential infectious trigger in AHOI pathophysiology.

## Introduction

Human metapneumovirus (hMPV), a respiratory pathogen, was first identified in 2001 [1]. It can cause a range of illnesses, from asymptomatic carrier states to severe bronchiolitis. Children aged six and under make up 96% of those testing positive for hMPV [1]. Beyond encephalitis, hMPV has also been associated with acute otitis media in children [2]. While hMPV infections can occur throughout the year, their incidence peaks during winter and spring, coinciding with the seasons for respiratory syncytial virus (RSV) and seasonal influenza [3,4].

Acute Haemorrhagic Oedema of Infancy (AHOI), also known as Acute Hemorrhagic Edema of Infancy (AHEI), is a rare form of cutaneous leukocytoclastic vasculitis, primarily affecting infants and toddlers. The condition typically presents with peripheral edema and characteristic targetoid purpuric lesions, particularly on the face and extremities [5]. AHOI is most frequently observed in boys between 4 and 24 months of age and does not show any racial predilection [5]. Irving M. Snow first described AHOI in the United States in 1913, classifying it as a cutaneous variant of Henoch-Schönlein purpura (HSP) [6].

The etiology of AHOI is not completely understood, but it is thought to involve immune complex deposition in small blood vessels, which triggers inflammatory and hemorrhagic responses in the skin. This mechanism suggests that infections, medications, or vaccinations may act as precipitating factors, possibly due to an overactive immune response in young children. Infections are a prominent factor, with AHOI previously being linked to a variety of pathogens, including rotavirus [7], cytomegalovirus (CMV) [5], and pneumococcal bacteremia [8].

Epidemiologically, the organisms associated with AHOI have variable prevalence. For instance, rotavirus infections are among the more frequently reported causes, with a higher prevalence in regions with limited vaccination uptake [7]. CMV is a less common association, observed primarily in immunocompromised individuals or through vertical transmission [5]. Pneumococcal bacteremia has also been identified as an etiologic agent but remains relatively rare due to widespread vaccination practices [8]. To date, only two cases of AHOI have been linked to hMPV infection, highlighting its rarity as a causative organism. The potential for hMPV to contribute to AHOI pathophysiology is plausible, given the inflammatory response associated with respiratory pathogens, which may lead to immune complex deposition in skin vasculature, mirroring mechanisms seen in other infectious triggers.

## Case presentation

**Figure 1:**
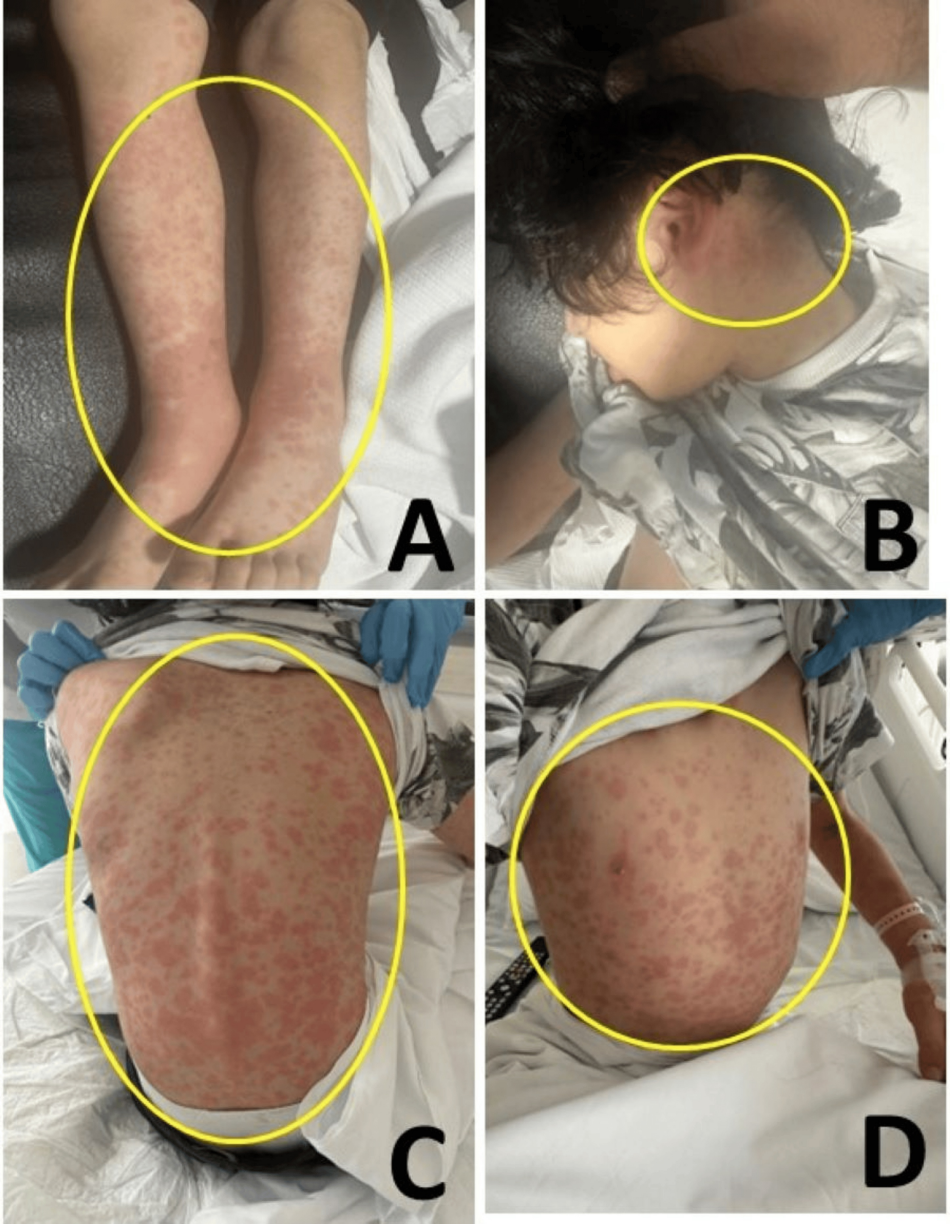
Rash and purpura seen at different areas of body Panel (A) shows rash and purpura at both the lower extremities; Panel (B) shows rash and purpura near the neck region; Panels (C) and (D) show rash and purpura on the back from different angles.

An eight-year-old boy presented to the emergency room two days ago with a widespread, non-blanching red macular rash that covered his face, chest, abdomen, back, and legs, as shown in Figure 1. Throughout the course of his illness, the rash progressively increased in size. In addition to the rash, he experienced a mild fever and coryzal symptoms. He had no history of allergies and was fully immunized. Upon examination, the rash was observed on the child’s face, chest, abdomen, back, and lower limbs, as well as on the palms and soles. The rash was warm to the touch, well-defined, and irregularly shaped. There were no nodules, vesicles, pustules, or target lesions present. Furthermore, the mucous membranes, including the oral and vaginal areas, were unaffected.

The child experienced severe edema in both hands, feet, and ankles, leading to significant swelling that impaired his ability to walk normally. Aside from this, his systemic examination was unremarkable. Clinical findings suggested that the rash and edema indicated AHOI. A comprehensive blood panel included liver function tests, coagulation profiles, C-reactive protein (CRP), and iron levels. The results are summarized in Table 1. The white blood cell count was normal, but there was an elevated neutrophil count and increased CRP levels (70 mg/L). Hypochromic, microcytic erythrocytes were noted, along with the presence of toxic granules in some polymorphonuclear cells. All other blood test results were within normal reference ranges. hMPV was detected in a nasopharyngeal aspirate.

**Table 1:** Blood test results and reference ranges

Test	Result	Reference range
White blood cell (WBC) count	Normal	4,000-11,000 cells/µL
Neutrophil count	Elevated	40-60% of total WBC
C-reactive protein (CRP)	70 mg/L	< 5 mg/L
Erythrocyte morphology	Hypochromic, microcytic	Normal: normochromic, normocytic
Toxic granules	Present in some polymorphs	Not typically present

**Figure 2:**
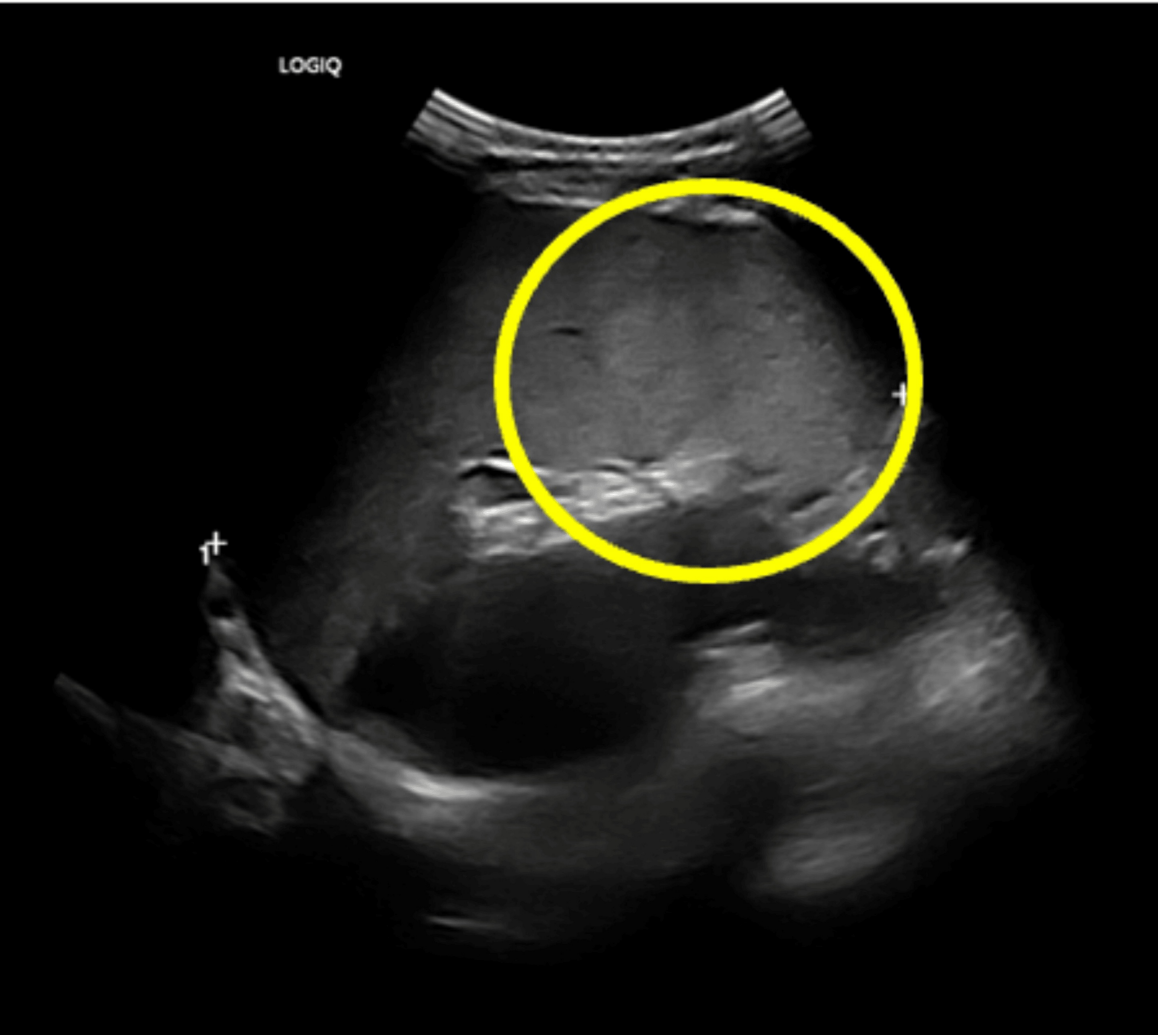
Abdominal ultra sonography The marked region shows splenomegaly.

An abdominal ultrasound revealed moderate splenomegaly and enterocolitis, as shown in Figure 2. The rash rapidly subsided over the next two days, coinciding with a marked reduction in lower limb edema. His pain was managed with non-steroidal anti-inflammatory drugs and antihistamines.

## Discussion

AHOI is a rare form of leukocytoclastic vasculitis primarily affecting children between four months and two years of age [5]. The condition is distinguished by its cutaneous manifestations - large, erythematous purpuric lesions typically appearing on the face and extremities. AHOI was previously considered a variant of HSP but is now recognized as a distinct condition [9]. HSP remains a key differential diagnosis, particularly as it predominantly affects older children, typically aged four to seven years [9]. Clinically, HSP differs in its presentation, with smaller, palpable purpura primarily distributed over the legs and buttocks [10]. Table 2 provides a summary contrasting the clinical features of AHOI and HSP for diagnostic clarity.

**Table 2:** Key differences between AHOI and HSP AHOI: Acute Haemorrhagic Oedema of Infancy; HSP: Henoch-Schönlein Purpura; GI: Gastrointestinal

Feature	AHOI	HSP
Age group	4 months to 2 years	4 to 7 years
Skin lesions	Large, erythematous purpura on face, extremities	Palpable purpura on legs, buttocks
Visceral involvement	Rare	Common (renal, GI)
Immunopathology	Leukocytoclastic vasculitis	IgA-mediated vasculitis
Common triggers	Infection, medications	Often idiopathic

In this case, hMPV was detected, which is rarely associated with AHOI. This virus may have contributed to the pathophysiology through immune complex formation and deposition in the skin's microvasculature, triggering a localized inflammatory response. The potential mechanism linking hMPV to AHOI could involve immune response pathways similar to those seen in other infections associated with AHOI, such as CMV and rotavirus. Specifically, hMPV is known to stimulate both innate and adaptive immune responses, which might lead to excessive cytokine release and immune complex deposition, creating an environment conducive to vasculitis. The presence of splenomegaly in this case adds an interesting clinical dimension. Although splenomegaly is not typically associated with AHOI, it may represent an atypical response to hMPV infection or a broader systemic immune reaction. Splenomegaly could reflect underlying immune hyperactivity or a response to systemic inflammation, as seen in some viral infections. Further studies are needed to clarify the significance of splenomegaly in the context of AHOI and hMPV.

Approximately 75% of AHOI cases are preceded by an infection, most commonly respiratory infections, suggesting a potential hypersensitivity or immune-mediated reaction to pathogens. Various microorganisms have been associated with AHOI, including streptococci, staphylococci, adenovirus, and CMV, among others [10]. In addition to infections, vaccinations and certain medications have been reported as triggers, supporting an immune complex hypersensitivity mechanism in the etiology of AHOI [11]. The diagnostic workup for AHOI may include laboratory tests, such as a complete blood count, which can reveal nonspecific findings like leukocytosis or thrombocytosis, and inflammatory markers, including elevated erythrocyte sedimentation rate (ESR) and CRP [10]. In this case, neutrophilia and elevated CRP levels suggest an ongoing inflammatory process, potentially heightened by hMPV infection. Histological examination of AHOI lesions, if performed, typically reveals perivascular neutrophilic infiltration with nuclear fragmentation and fibrinoid necrosis, consistent with leukocytoclastic vasculitis [10]. Although AHOI is generally benign, serious complications are possible, including renal involvement, gastrointestinal hemorrhage, epididymo-orchitis, and cartilage damage [10].

Treatment and emerging strategies

Management of AHOI is primarily supportive, as the condition is self-limiting in most cases. In this case, treatment was conservatively managed, aligning with established guidelines that recommend supportive care without routine corticosteroid administration, as corticosteroids are generally ineffective for AHOI [11]. Supportive care included the administration of antipyretics, such as acetaminophen, at a dosage of 15 mg/kg every six hours as needed for fever management, and intravenous fluids to maintain hydration, typically at a rate of 10-20 mL/kg/hour, adjusted based on clinical needs. The treatment duration for supportive care generally depends on the clinical course of the condition, with monitoring conducted over several days until the child’s condition stabilizes and symptoms resolve [12].

Although antiviral therapies are not standard for AHOI, emerging treatments for hMPV, including monoclonal antibodies and antiviral agents, are under investigation and may benefit immunocompromised patients with hMPV infections [13-15]. Such advancements underscore the potential for novel approaches in managing hMPV infections, which may indirectly influence cases where hMPV is associated with rare conditions like AHOI.

## Conclusions

In conclusion, hMPV, a respiratory pathogen first identified in 2001, primarily affects young children and typically presents with symptoms ranging from mild respiratory illness to severe bronchiolitis and acute otitis media, particularly during winter and spring. AHOI is a rare leukocytoclastic vasculitis seen predominantly in infants, with symptoms of peripheral edema and targetoid purpuric lesions. Although AHOI has been associated with infections like rotavirus, CMV, and pneumococcal bacteremia, its occurrence alongside hMPV is exceedingly rare. This case underscores a previously undocumented complication of hMPV, contributing to the growing medical understanding of its broader clinical spectrum. Recognizing this potential association may aid in the early identification and management of similar cases, highlighting the need for increased awareness and further research into hMPV’s role in immune-mediated conditions such as AHOI.

## References

[1] Domachowske J, Russell WS (2013 (2024). Paediatric human metapneumovirus. https://emedicine.medscape.com/article/972492-overview.

[2] Suzuki A, Watanabe O, Okamoto M, Endo H, Yano H, Suetake M, Nishimura H (2005). Detection of human metapneumovirus from children with acute otitis media. Pediatr Infect Dis J.

[3] Schildgen O, Glatzel T, Geikowski T (2005). Human metapneumovirus RNA in encephalitis patient. Emerg Infect Dis.

[4] Schweon SJ (2013). Human metapneumovirus. Nursing.

[5] Kuroda K, Yabunami H, Hisanaga Y (2002). Acute haemorrhagic oedema of infancy associated with cytomegalovirus infection. Br J Dermatol.

[6] Snow IM (1913). Purpura, urticaria, and angioneurotic oedema of the hands and feet in a nursing baby. JAMA.

[7] Di Lernia V, Lombardi M, Lo Scocco G (2004). Infantile acute hemorrhagic edema and rotavirus infection. Pediatr Dermatol.

[8] Morrison RR, Saulsbury FT (1999). Acute hemorrhagic edema of infancy associated with pneumococcal bacteremia. Pediatr Infect Dis J.

[9] Scheinfeld N (2009). Dermatology. Dermatol Online J.

[10] Shah D, Goraya JS, Poddar B, Parmar VR (2002). Acute infantile hemorrhagic edema and Henoch-Schönlein purpura overlap in a child. Pediatr Dermatol.

[11] Shenenberger D, James WD (2018 (2024). Acute hemorrhagic edema of infancy. https://emedicine.medscape.com/article/1112590-overview.

[12] Feng Y, He T, Zhang B, Yuan H, Zhou Y (2024). Epidemiology and diagnosis technologies of human metapneumovirus in China: a mini review. Virol J.

[13] Cucinotta U, Mazza F, Pajno GB, Gallizzi R (2020). Acute haemorrhagic oedema of infancy: a condition that is not always benign. BMJ Case Rep.

[14] Deval H, Kumar N, Srivastava M (2024). Human metapneumovirus (hMPV): an associated etiology of severe acute respiratory infection in children of Eastern Uttar Pradesh, India. Access Microbiol.

[15] Veronese A, Uršič T, Bizjak Vojinovič S, Rodman Berlot J (2024). Exploring clinical predictors of severe human metapneumovirus respiratory tract infections in children: insights from a recent outbreak. Microorganisms.

